# The splicing factor DHX38/PRP16 is required for ovarian clear cell carcinoma tumorigenesis, as revealed by a CRISPR‐Cas9 screen

**DOI:** 10.1002/2211-5463.13358

**Published:** 2022-01-28

**Authors:** Brandon Cona, Tomoatsu Hayashi, Ai Yamada, Naomi Shimizu, Naoko Yokota, Ryuichiro Nakato, Katsuhiko Shirahige, Tetsu Akiyama

**Affiliations:** ^1^ Laboratory of Molecular and Genetic Information Institute of Quantitative Biosciences The University of Tokyo Japan; ^2^ Laboratory of Computational Genetics Institute of Quantitative Biosciences The University of Tokyo Japan; ^3^ Laboratory of Genome Structure and Function Institute of Quantitative Biosciences The University of Tokyo Japan

**Keywords:** apoptosis, DHX38, ovarian cancer, PRP16, splicing factor, tumorigenesis

## Abstract

Certain cancers, such as ovarian clear cell carcinoma (OCCC), display high levels of genetic variation between patients, making it difficult to develop effective therapies. In order to identify novel genes critical to OCCC growth, we carried out a comprehensive CRISPR‐Cas9 knockout screen against cell growth using an OCCC cell line and a normal ovarian surface epithelium cell line. We identified the gene encoding DHX38/PRP16, an ATP‐dependent RNA helicase involved in splicing, as critical for the growth and tumorigenesis of OCCC. *DHX38/PRP16* knockdown in OCCC cells, but not normal cells, induces apoptosis and impairs OCCC tumorigenesis in a mouse model. Our results suggest that DHX38/PRP16 may play a role in OCCC tumorigenesis and could potentially be a promising therapeutic target.

## Graphical abstract

The development of effective therapies against ovarian clear cell carcinoma is complicated by its lack of an overarching mutational signature. By employing a CRISPR‐Cas9 screen, we uncovered DHX38/PRP16 as a novel genetic vulnerability whose knockdown results in growth suppression of ovarian clear cell carcinoma both *in vitro* and *in vivo*.

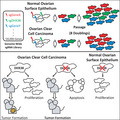

**Abbreviations**
ARID1A: AT‐rich interaction domain 1A
BAF: brg1‐ or bgm‐associated factors
CRISPR: clustered regularly interspaced short palindromic repeat
DHX38/PRP16: DEAH‐box helicase 38/pre‐mRNA‐splicing factor ATP‐dependent RNA helicase PRP16
GADD45A: growth arrest and DNA damage‐inducible alpha
GeCKO: genome‐scale CRISPR knock‐out
MAGeCK: model‐based analysis of genome‐wide CRISPR‐Cas9 knockout
OCCC: ovarian clear cell carcinoma
OSE: ovarian surface epithelium
PIK3CA: phosphatidylinositol‐4,5‐bisphosphate 3‐kinase catalytic subunit alpha
PP4: protein phosphatase 4
PUMA: p53 upregulated modulator of apoptosis
sgRNA: single‐guide RNA
SWI/SNF: switch/sucrose nonfermentable

Epithelial ovarian cancer is divided into five major histological subtypes. Of these, the clear cell subtype [ovarian clear cell carcinoma (OCCC)] shows particularly poor prognoses, with higher stage OCCC tumors presenting with severely low 5‐year survival rates of between 20% and 30% [1, 2]. A major factor underlying these poor prognoses is the poor response (1–8%) OCCC tumors exhibit against conventional platinum‐based therapies [3, 4].

Ovarian clear cell carcinoma is characterized by frequent mutations in AT‐rich interaction domain 1A (ARID1A), a member of the BAF subclass of the human switch/sucrose nonfermentable (SWI/SNF) complex which acts modulate histone accessibility [5, 6]. Frequent mutations are also observed in phosphatidylinositol‐4,5‐bisphosphate 3‐kinase catalytic subunit alpha (PIK3CA), which promotes tumor proliferation and survival through the phosphorylation of downstream factors [7, 8]. Mutations in ARID1A and PIK3CA frequently co‐occur and are considered to cooperate in OCCC tumorigenesis. However, aberrations in the PI3K/AKT/mTOR pathway and SWI/SNF complex alone are insufficient to fully describe the mechanisms underlying OCCC tumorigenesis in the majority of cases, confounding the development of precision therapies, whose applicability is often based on the presence of specific sensitizing mutations.

In recent years, clustered regularly interspaced short palindromic repeat (CRISPR)‐Cas9 has come to prominence as the leading tool for *in vitro* and *in vivo* gene editing. By employing designer single guide RNAs (sgRNAs), double‐strand breaks can be introduced almost anywhere in the genome; by taking advantage of the cell's innate repair machinery one can not only introduce mutations, but also whole expression cassettes and even large deletions. This has led to the development of a number of pooled screening approaches utilizing the CRISPR‐Cas9 system to screen for genes critical to a number of cellular processes [9, 10, 11].

In the current study, we employed a CRISPR‐Cas9‐based screening system to identify novel genes critical to OCCC cell growth in order to better understand the mechanisms underlying OCCC tumorigenesis and help aid the development of novel therapies against it. We show that DEAH‐box helicase 38 (DHX38/PRP16), known for its role in priming the spliceosomal C complex for exon ligation through a rearrangement allowing for 3′‐splice site docking during mRNA splicing [12, 13, 14, 15, 16], plays a critical role in the proliferation and tumorigenesis of OCCC.

## Materials and methods

### Ethical statement

Mouse experiments were approved by the Ethics Committee of the Institute of Quantitative Bioscience, The University of Tokyo, and were performed according to ‘the Guidelines for Proper Conduct of Animal Experiments’ provided by the Science Council of Japan.

### Cell culture

Cell lines and composition of culture media can be found in Table 1. All cell lines were cultured at 37 °C under a 5% CO_2_ atmosphere.

**Table 1:** Cell lines and culture media composition.

Cell line name	Tissue of origin	T‐antigen immortalized?	Culture Medium			FBS
			Medium name	Manufacturer	Catalog #	
OSE1	OSE	Yes	RPMI1640	Nissui	#5918	10%
OSE3	OSE	Yes	RPMI1640	Nissui	#5918	10%
JHOC5	Ovarian clear cell carcinoma	No	RPMI1640	Nissui	#5918	10%
ES2	Ovarian clear cell carcinoma	No	McCoy's 5A	Sigma	#M4892	10%
TOV21G	Ovarian clear cell carcinoma	No	MCDB 105 (50%)/Medium 109 (50%)	Sigma/Sigma	#M6395 /#M2520	15%
HEK293FT	Embrionic kidney cells	Yes	DMEM	Nissui	#5919	10%
HCT116	Colorectal carcinoma	No	McCoy's 5A	Sigma	#M4892	10%

### Growth assays

Cell growth was assayed using the Cell‐Titer Glo Luminescent Cell Viability Assay (Promega, Madison WI, USA, #G7573) following the manufacturer's protocol. Plates were read on a Berthold Mithras LB 940 Plate Reader.

### Viral construction

The Human CRISPR Knockout Pooled Library (GeCKO v2) [17] comprising the lentiCas9‐Blast and lentiGuide‐Puro two vector system was purchased from Addgene (Watertown MA, USA, #1000000049) and prepared following the manufacturer's instructions. shRNA vectors (Table 2) were inserted into the CS‐Rfa‐CG backbone (provided by H. Miyoshi, RIKEN BioResource Research Center, Tsukuba, Japan). Lentiviruses were prepared via cotransfection of 1 × 10^7^ HEK293FT cells per 10 cm culture plate with the appropriate vector along with the packaging plasmids psPAX2 (Addgene #12260) and pMD2.G (Addgene #12259) in a 2 : 1 : 1 ratio (15 μg plasmid DNA per plate) using 60 μL of 1 mg·mL^−1^ polyethylenimine ‘MAX’ (Polysciences, Inc., Warrington PA, USA, #24765) in a 4 : 6 mix of Opti‐MEM reduced serum medium (Gibco, Waltham MA, USA, #22600050):Dulbecco's Modified Eagle Medium (DMEM; Nissui, Tokyo, Japan, #5919). Lentiviral particles were collected via ultracentrifugation in an Optima XE‐90 Ultracentrifuge at an RCF of 76,800 x *g* (r_avg_) / 106,000 x *g* (r_max_)  72 h post‐transfection and reconstituted in PBS for at least 24 h before use in downstream experiments.

**Table 2:** shRNA sequences used in RNAi experiments.

shRNA name	shRNA Sequence (SENSE‐loop‐ANTISENSE)
shLuc	GATTTCGAGTCGTCTTAAATGTgcttcctgtcacACATTTAAGACGACTCGAAATC
shDHX38/PRP16#1	GAAGGAATTTCATTTGACACGgcttcctgtcacCGTGTCAAATGAAATTCCTTC
shDHX38/PRP16#2	GATCACATGAAGAGAAAGAGCgcttcctgtcacGCTCTTTCTCTTCATGTGATC

### CRISPR‐Cas9 screening

JHOC5 and ovarian surface epithelium (OSE) cells were infected with the GeCKO v2 two‐vector system as described previously [18]. Briefly, JHOC5‐Cas9 and OSE3‐Cas9 expression cell lines were established by lentiviral transfection of lentiCas9‐Blast (Addgene #52962) at an multiplicities of infection (MOI) of 0.3 followed by selection with blasticidin. These cell lines were then further transfected with a lentivirus containing the lentiGuide‐Puro A library (Addgene # 1000000049) at an MOI of 0.3. Following this transfection step, cells were maintained in an appropriate concentration of puromycin. One day after infection, a sample of 2 × 10^7^ infected cells (back‐calculated from the MOI) was pelleted and frozen under liquid nitrogen as a Day 0 sample. Cells were then passaged until they had undergone 8 doublings. To maintain sufficient sgRNA coverage, the total number of cells was maintained above 2 × 10^7^ for the duration of the culture period. A second sample of 2 × 10^7^ cells was taken as a final time point. Genomic DNA was extracted from the above‐mentioned samples using a QIAGEN Blood and Cell Culture DNA Midi Kit (QIAGEN, Germantown MD, USA, #13343). 125 μg of genomic DNA from each sample was split into 2.5 μg fractions, and sgRNA sequences were amplified to form a sequencing library as described previously [18] in two steps, using the primers in Table 3. Reactions were measured for fragment size using the Agilent 2200 Tapestation and quantified using the KAPA SYBR Fast qPCR Kit (KAPA Biosystems, Wilmington MA, USA, #7959362001). Libraries were sequenced on an Illumina HiSeq2500.

**Table 3:** Primers used to amplify sgRNA sequences.

First PCR primers	
Forward:	AATGGACTATCATATGCTTACCGTAACTTGAAAGTATTTCG
Reverse:	TCTACTATTCTTTCCCCTGCACTGTTGTGGGCGATGTGCGCTCTG
Second PCR Primers	
Forward #06:	AATGATACGGCGACCACCGAGATCTACACTCTTTCCCTACACGACGCT CTTCCGATCTATCGATTCTTGTGGAAAGGACGAAACACCG
Forward #08:	AATGATACGGCGACCACCGAGATCTACACTCTTTCCCTACACGACGCT CTTCCGATCTCGATCGATTCTTGTGGAAAGGACGAAACACCG
Forward #09:	AATGATACGGCGACCACCGAGATCTACACTCTTTCCCTACACGACGCT CTTCCGATCTACGATCGATTCTTGTGGAAAGGACGAAACACCG
Forward #10:	AATGATACGGCGACCACCGAGATCTACACTCTTTCCCTACACGACGCT CTTCCGATCTTTCTTGTGGAAAGGACGAAACACCG
Reverse #01:	CAAGCAGAAGACGGCATACGAGATCGTGATGTGACTGGAGTTCAGAC GTGTGCTCTTCCGATCTTCTACTATTCTTTCCCCTGCACTGT
Reverse #03:	CAAGCAGAAGACGGCATACGAGATTCAAGTGTGACTGGAGTTCAGAC GTGTGCTCTTCCGATCTTCTACTATTCTTTCCCCTGCACTGT
Reverse #05:	CAAGCAGAAGACGGCATACGAGATAAGCTAGTGACTGGAGTTCAGAC GTGTGCTCTTCCGATCTTCTACTATTCTTTCCCCTGCACTGT
Reverse #07:	CAAGCAGAAGACGGCATACGAGATGGCCACGTGACTGGAGTTCAGAC GTGTGCTCTTCCGATCTTCTACTATTCTTTCCCCTGCACTGT

### RNAi experiments

For siRNA experiments, Lipofectamine RNAiMAX Transfection Reagent (Thermo Fisher, Waltham MA, USA, #13778500) was used according to the manufacturer's protocol to transfect siRNA oligomers purchased from Ambion (Austin TX, USA, siCtrl#1 ‐ #4390844, siDHX38/PRP16#1 ‐ s18906, siDHX38/PRP16#2 ‐ s18908) into dissociated cells at a concentration of 10 nm for siRNAs and 0.167% v/v for RNAiMAX reagent. For shRNA experiments, reconstituted lentiviral particles were added to dissociated cells suspended in PBS, topped to 1 mL with PBS, and allowed to incubate for 1 h at 37 °C before plating. Cells were infected at the following MOI: JHOC5 ‐ 25, OSE1, TOV21G, and ES2 ‐ 50, OSE3 ‐ 100, with titers determined using TOV21G.

### Subcutaneous xenografts

After being trypsinized and washed twice, once with culture medium followed by once with PBS, 1.5 × 10^7^ TOV21G cells or 1.0 × 10^6^ ES2 cells transferred to a 15 mL falcon tube (VIOLAMO, AS ONE, Osaka, Japan, #VIO‐15RN) were mixed with an appropriate volume of lentivirus suspension (MOI = 25 and 50), respectively, brought up to 1 mL with PBS, and allowed to incubate at 37 °C for 1 h. Infected cells were plated at an appropriate density (such that cells were no > 70% confluent 2 days after plating) onto 10 cm^2^ culture plates. Two days later, cells were trypsinized and washed three times with PBS. A sample of cells was reserved to confirm DHX38/PRP16 knockdown, and the rest were resuspended in 50% Matrigel at a concentration of 1.0 × 10^7^ cells·mL^−1^ for TOV21G cells and 6.67 × 10^6^ cells·mL^−1^ for ES2 cells. 150 μL of this suspension was injected into the flanks of female Balb/c nu/nu mice (Charles River Laboratories, Yokohama, Japan), and tumor volume was measured periodically.

### Western blot

Cells were harvested and lysed using a solution containing 50 mm Tris/HCl, 0.14 mm NaCl, 1 mm EDTA, and 1% NP‐40 along with 1x Halt Protease Inhibitor Cocktail (Thermo Fisher, #78438). Protein concentration was measured using the Pierce BCA Protein Assay (Thermo Fisher, #23225) as described by the manufacturer. Proteins were separated by SDS/PAGE and transferred to an Immobilion PVDF Membrane (Millipore, Burlington MA, USA, #IPVH00010) by electroblotting. All primary and secondary antibodies used can be found in Table 4. Protein bands were visualized using Luminata Forte Western HRP substrate (Millipore, #WBLUF0500).

**Table 4:** List of Antibodies used in western blotting.

Antibody name	Supplier/manufacturer	Catalog #	Dilution
Anti‐DHX38/PRP16	Proteintech	10098‐2‐AP	1 : 1000
Anti‐GAPDH	Millipore	MAB374	1 : 1000
Anti‐αTubulin	SantaCruz	sc32293	1 : 1000
Anti‐PUMA	Cell Signalling	12450	1 : 1000
Anti‐TP53	SantaCruz	sc126	1 : 1000
Anti‐Cleaved Caspase‐3	Cell Signalling	9664	1 : 1000
HRP‐linked ECL Sheep anti‐Mouse IgG	GE	NA931V	1 : 5000
HRP‐linked ECL Donkey anti‐Rabbit IgG	GE	NA934V	1 : 5000

### RNA extraction and qRT‐PCR

Total RNA was extracted from at least 1 × 10^5^ cells and purified using TRIsure (Meridian Bioscience, Cincinnati OH, USA, #BIO‐38033) following the manufacturer's protocol. Synthesis of cDNA was carried out using Prime Script RT Master Mix (TaKaRa, Kusatsu, Saga Prefecture, Japan, #RR036B) following the manufacturer's instructions. An amount of synthesized cDNA corresponding to 10 ng of original RNA was mixed with primers (Table 5) each at a final concentration of 1 μm and 5 μL of 2× SYBR Green Master Mix (Thermo Fisher, #4334973) in a final volume of 10 μL and measured on a LightCycler480 (Roche, Basel, Switzerland). The ΔΔCT method was used to relatively quantify concentrations, with GAPDH used as an internal control.

**Table 5:** qRT‐PCR primer sequences.

Gene		Sequence
GAPDH	forward:	GCACCGTCAAGGCTGAGAAC
	reverse:	TGGTGAAGACGCCAGTGGA
DHX38/PRP16	forward:	GCGGGATAGAAGTAGGCACAG
	reverse:	GAAGGGGTGGCTGCATCTTTA
PUMA	forward:	GACCTCAACGCACAGTACGA
	reverse:	AGGACCCTCCAGGGTGAG
GADD45A	forward:	GCCAAGCTGCTCAACGTC
	reverse:	CTCTGTCGTCGTCCTCGTC

### Sub‐G1 assays

2 × 10^5^ cells per well of a six‐well plate were infected with shRNA‐containing lentiviruses (as described in the xenograft experiment section) and cultured for 4 days. Cells were then trypsinized, collected, and fixed in a 70% ethanol:water solution at −30 °C overnight. The following day, cells were incubated in a 4 mm citric acid (pH 8.0), 200 mm Na_2_HPO_4_ solution for 20 min, stained with a solution of 10 μg·mL^−1^ propidium iodide (Sigma, #P4170), and 10 μg·mL^−1^ RNase A (Sigma, Burlington MA, USA, RNASEA‐RO) in 1× PBS for an additional 20 min, and subsequently analyzed on a Sony EC800 Flow Cytometry Analyzer (Sony Biotechnology, San Jose CA, USA).

### Data and statistical analyses

**Fig. 1:**
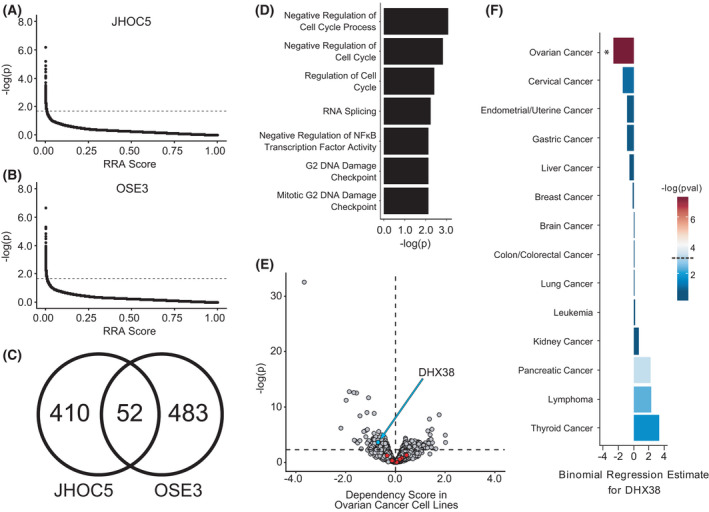
CRISPR‐Cas9 screening results. (A, B) MAGeCK was used to identify genes whose targeting sgRNAs were significantly and consistently depleted over the course of the culture period for JHOC5 (A) and OSE3 (B) cells (points represent genes; dotted line indicates *P* = 0.02). (C) Venn diagram comparing the numbers of significantly depleted genes in JHOC5 and OSE3 cells. (D) Results of GO statistical overrepresentation analysis of JHOC5‐specific hit genes using the GO‐Slim Biological Process gene set. Only significantly (*P* < 0.01) overrepresented pathways are displayed. Differential dependency scores relative to epithelial ovarian cancer cell lines. Points indicate genes. Colored points (red and cyan) indicate candidate genes. Cyan points represent candidate genes with an ovarian cancer‐specific differential dependency of < 0 and a *P*‐value of ≤ 0.0005. Red points represent candidate genes that do not fit these criteria. The vertical dotted line represents an ovarian cancer‐specific dependency score of 0.0; the horizontal dotted line represents a *P*‐value of 0.0005. (F) Logistic regression results of DHX38/PRP16 dependency scores in cell lines of various lineages. Dependency scores for each indicated lineage vs all cell lines were modeled as a function of lineage. The *P*‐value for each regression is indicated by the color of each individual bar (*P < 0.001; dotted line on figure legend represents *P* = 0.001).

**Fig. 2:**
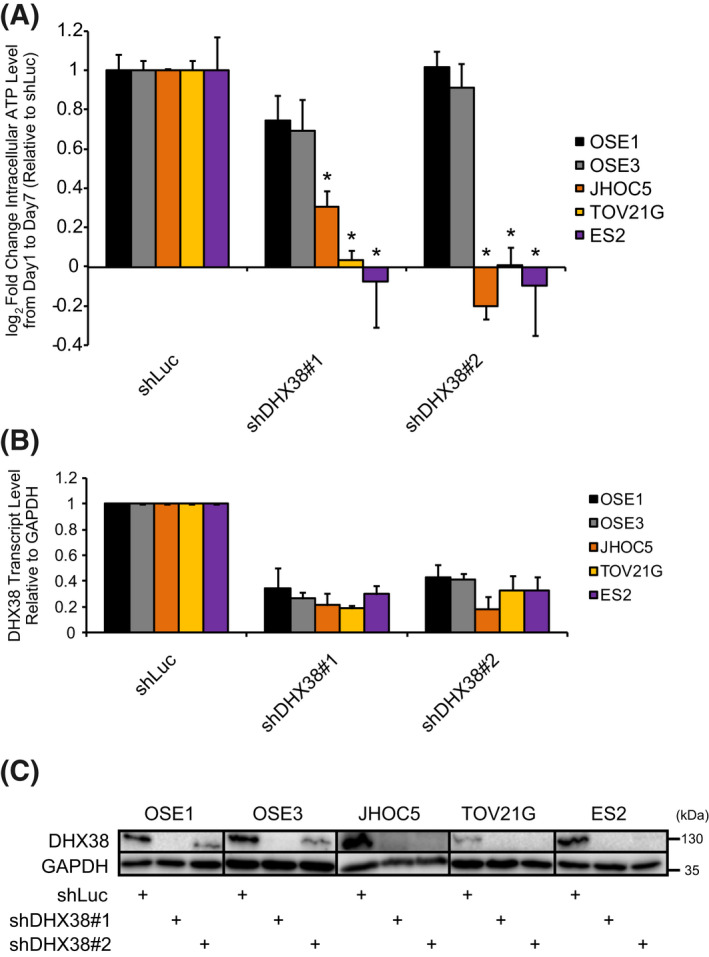
DHX38/PRP16 knockdown results in growth inhibition of OCCC cell lines. (A) Fold change in intracellular ATP level from days 1 ro 7 postinfection with an shDHX38/PRP16‐containing lentivirus, normalized to shLuc (*P < 0.05 compared to both OSE1 and OSE3; unpaired *t*‐test; error bars represent SD; all data *n* = 3). (B) DHX38/PRP16 shRNA knockdown efficiencies measured 4 days postinfection, normalized to shLuc. GAPDH was used as an internal control. (error bars represent SD; OSE1, OSE3, JHOC5, TOV21G, *n* = 3; ES2, *n* = 4) (C) Western blot of DHX38/PRP16 4 days postinfection (*n* = 2, representative data shown).

**Fig. 3:**
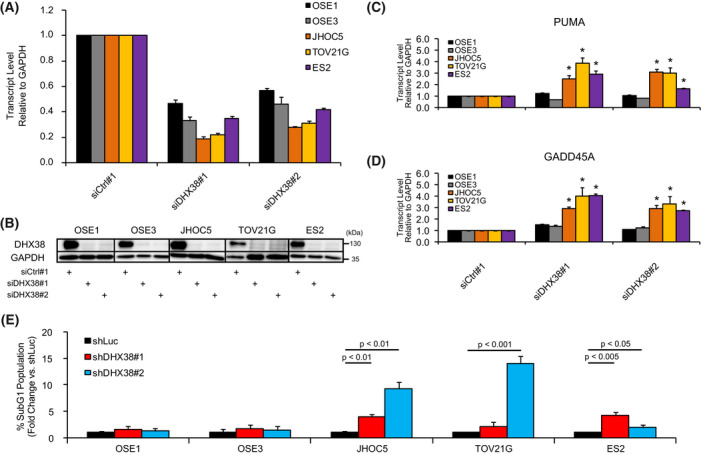
Knockdown of DHX38/PRP16 induces apoptosis in OCCC cells. (A) Confirmation of siRNA‐mediated DHX38/PRP16 knockdown, normalized to an siRNA control sequence. GAPDH was used as an internal control. (error bars represent SD; all data *n* = 3) (B) Western blot of DHX38/PRP16 4 days post‐siRNA‐transfection. (*n* = 2, representative data shown) (C, D) Effect of siRNA‐mediated DHX38/PRP16 knockdown on PUMA (C) and GADD45A (D) transcript levels in the indicated cell lines, normalized to a control siRNA. GAPDH was used as an internal control. (error bars represent SD; *P < 0.05 compared to both OSE1 and OSE3; unpaired Student's *t*‐test; all data *n* = 3). (E) Relative Sub‐G1 cell populations 4 days postinfection with shRNA‐containing lentiviruses (*P*‐values obtained using unpaired *t*‐test; error bars represent SD; all data *n* = 3).

**Fig. 4:**
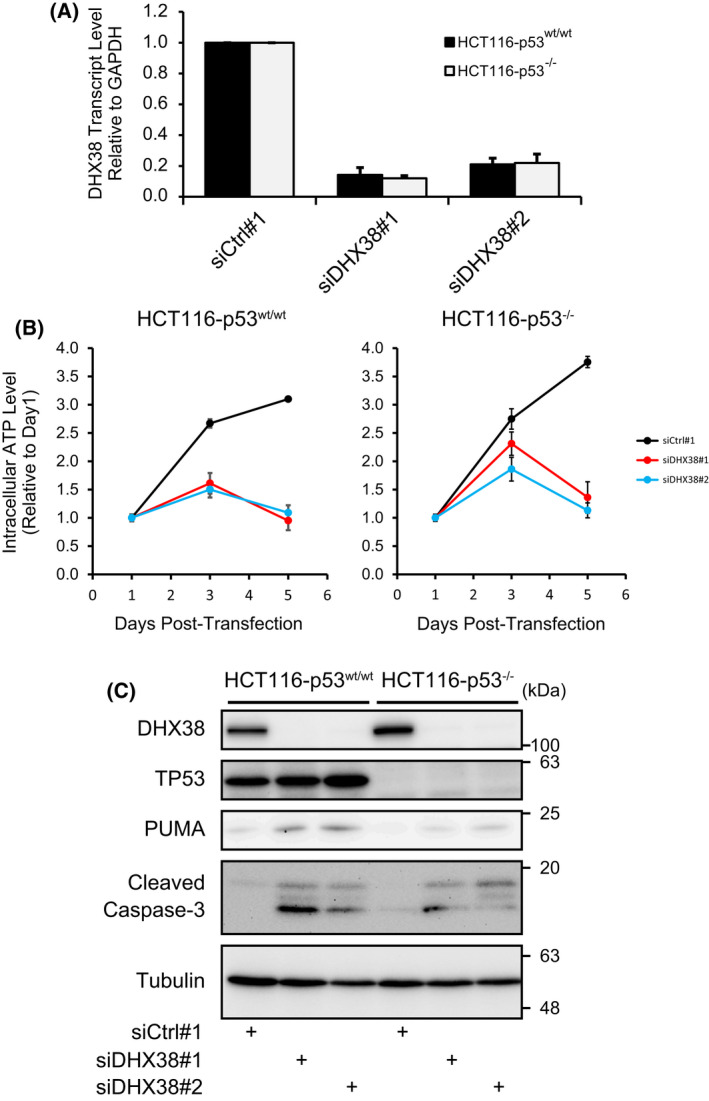
Response of HCT116‐p53^wt/wt^ and HCT116‐p53^−/−^ cells to DHX38/PRP16 knockdown. (A) Confirmation of siRNA‐mediated DHX38/PRP16 knockdown, normalized to a control siRNA sequence. GAPDH was used as an internal control. (error bars represent SD; *n* = 3) (B) Fold change in intracellular ATP level from days 1 to 5 post‐siRNA‐transfection, normalized to a control siRNA (results are shown as the average of 6 wells ± SD) (C) Western blot of DHX38/PRP16 2 days post‐siRNA‐transfection. (*n* = 2, representative data shown).

**Fig. 5:**
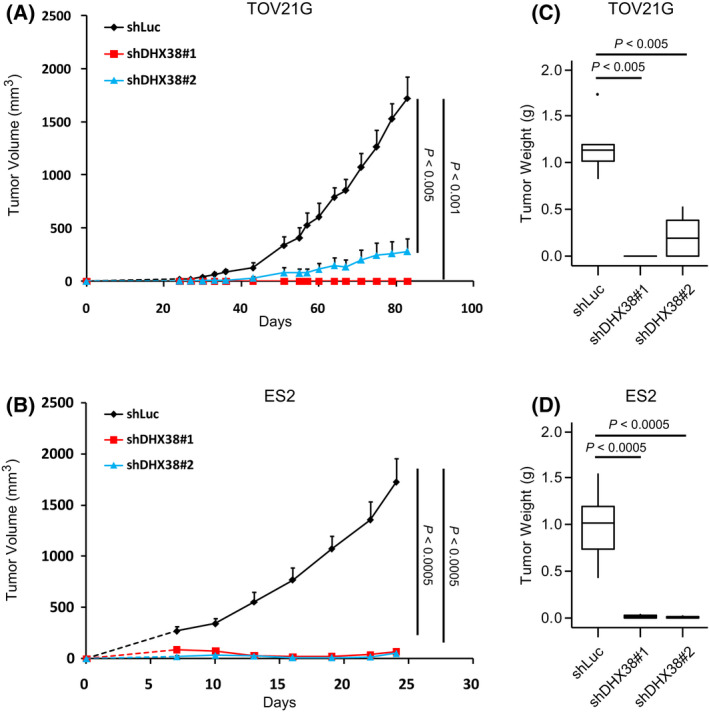
DHX38/PRP16 knockdown results in impaired tumor formation ability in OCCC cell lines. (A, B) Tumor volume of Balb/c nu/nu mice injected subcutaneously with TOV21G (A) or ES2 (B) cells expressing either shLuc or one of two shRNAs against DHX38/PRP16 (error bars represent SE; A, *n* = 5; B, *n* = 8; unpaired Student's *t*‐test, unequal variances). (C, D) After the end of the study (A, B), TOV21G (C) and ES2 (D) tumors were removed, sectioned, and weighed (error bars represent SE; unpaired Student's *t*‐test).

Statistical analyses, including Student's *t*‐tests, were performed using r version 3.6.0 (http://www.r‐project.org/). Plots in Figs 2, 3, 4 and 5A,B were generated in excel. Figure 1A,B was generated from the model‐based analysis of genome‐wide CRISPR‐Cas9 knockout (mageck) software. All other plots were generated in R using the ggplot2 package in conjunction with the cowplots package. The MAGeCK algorithm was carried out as published in Li et al. [19]. Briefly, sgRNAs ranked based on their abundance in cultured samples compared to controls were grouped by gene and assessed for skew to lower abundancies using the MAGeCK test command on the default settings. For detailed commands employed during the MAGeCK algorithm, as well as those used to generate of sgRNA count data, refer to GSE188485 (https://www.ncbi.nlm.nih.gov/geo/query/acc.cgi?acc=GSE188485). GO statistical overrepresentation analyses were carried out using the Panther Classification System website using the default Homo Sapiens Reference List (default) as a reference list. Differential dependencies for the Broad Institute dataset were calculated as in Meyers et al. [20] using the data.table package along with base functions. Briefly, the difference in mean between the gene dependencies of each epithelial ovarian cancer cell line and those of all other cell lines was calculated for each indicated gene. Significance was assessed via a two‐sided Student's *t*‐test with unequal variances. Logistic regression was carried out using the R stats package on the Broad Institute dataset using DHX38/PRP16 dependency scores as the predictor variable and binary classification (1 or 0) into epithelial ovarian cancer or other cancer type as the response variable.

## Results

### CRISPR‐Cas9 screen identifies genes critical to OCCC proliferation

In order to screen for genes critical to the growth of OCCC, we employed the pooled genome‐scale CRISPR Knock‐Out (GeCKO) v2 2‐vector CRISPR‐Cas9 knockout screening system [17] using the OCCC cell line JHOC5, as well as the T‐antigen immortalized normal OSE cell line OSE3 as a negative control. After performing next‐generation sequencing on the resulting amplified libraries, we used the MAGeCK robust rank aggregation algorithm to aggregate sgRNA depletion data across multiple sgRNAs targeting the same gene [19]. This allowed us to identify genes whose sgRNAs were, on average, significantly depleted in each cell line over the course of the culture period (Fig. 1A,B).

Comparing screening results between JHOC5 and OSE3 cell lines, we were able to identify 462 genes whose targeting sgRNAs were significantly (*P* < 0.02) depleted in JHOC5 cells after the growth period, compared to 535 genes in OSE3 cells (Fig. 1C), 52 of which were common to both cell lines. As far as we are aware, this is the first study to date to perform a comprehensive genome‐wide CRISPR‐Cas9 screening against not only an OCCC cell line, but also an OSE cell line in tandem. This distinction is especially important, as the inclusion of a normal sample is critical for the identification of cancer‐specific lethalities and growth‐promoting mechanisms. Indeed, analysis of the 52 genes common to both JHOC5 and OSE3 using the GO‐slim biological function panther classification system ontologies [21, 22] revealed them to be significantly (FDR < 0.05) enriched with housekeeping genes related to ribosomal assembly (GO:0000027), tRNA metabolism (GO:0006399), and translation (GO:0006412).

Performing GO statistical overrepresentation analysis on the JHOC5‐specific growth‐critical genes, we were able to identify seven significantly enriched (*P* < 0.01) ontologies (Fig. 1D). As expected, the top ontologies were related to cell cycle deregulation. However, among the most significant ontologies was the RNA splicing gene set (GO:0008380). Although splicing has long been implicated in a wide variety of cancer types [23, 24], the role it plays in OCCC remains unclear. As such, we decided to focus our attention on this category for further analysis, obtaining a pooled list of 11 splicing‐related genes (Table 6).

**Table 6:** Differential dependency analysis results. The EOC cell lines used in the above comparison are as follows: 59M, A2780, BIN67, CAOV3, COV318, COV362, COV413A, COV434, COV504, COV644, EFO21, EFO27, ES2, HEYA8, JHOC5, JHOM1, JHOS2, JHOS4, KURAMOCHI, MCAS, OAW28, OCIC5X, ONCODG1, OV7, OV90, OVCAR5, OVCAR8, OVISE, OVK18, OVMANA, OVTOKO, PA1, PEA1, RMUGS, SCCOHT1, SKOV3, SNU8, SNU840, TO14, TOV112D, TOV21G, UWB1289.

Gene name	Differential dependency score	*P*‐value
DHX38/PRP16	−0.69386973	0.025973804
SF3A3	0.45340611	0.272765798
LSM3	−0.32994562	0.299789270
HNRNPL	0.24815368	0.453808526
TRA2A	0.18016505	0.560350797
RBM6	−0.12259776	0.667789620
WBP4	−0.13344305	0.711976995
SRSF3	−0.10136770	0.755566120
DBR1	−0.06126871	0.829622128
SNRPD3	0.04768180	0.946654331

To help narrow down our list of candidate genes, we turned to a recently published public dataset of CRISPR‐Cas9 growth screenings carried out and curated by the Broad Institute [20, 25]. Using an algorithm designed to remove confounding effects from copy number variation, a dependency score is calculated for each gene in each cell line. These scores are normalized so that a score of 0.0 represents a gene nonessential for growth in a particular cell line, and a score of −1.0 represents a gene highly essential for growth in a particular cell line. We first calculated the differential dependency score of each of the previously identified 11 candidate genes (Table 6) from the RNA splicing gene set in 42 epithelial ovarian cancer cell lines, compared to all other cancer cell lines. We next employed a Student's *t*‐test to determine which of these genes have a consistently lower dependency score among ovarian cancer cell lines. Our decision to group OCCC cell lines with those of the other epithelial ovarian cancer subtypes was based on both the small sample size of cell lines belonging to the OCCC subtype as well as the lack of other cancers with a general mutational profile similar to OCCC. The only gene found to be significantly enriched in ovarian cancer cell lines was the splicing factor DHX38/PRP16 (Fig. 1E). To help confirm this result, we carried out binomial regression on the dependency score data for DHX38/PRP16, using it as a predictor variable to model the likelihood of a cell line belonging to a particular lineage. We found that out of 14 clinically relevant lineages tested, only epithelial ovarian cancer was significantly (*P* < 0.001) likely to display a more negative DHX38/PRP16 dependency score compared to all other cell lines (Fig. 1F).

### DHX38/PRP16 knockdown inhibits growth of OCCC, but not OSE

We first sought to ensure that the DHX38/PRP16 growth dependency observed in JHOC5 cells was not JHOC5‐specific by employing RNAi to knockdown DHX38/PRP16 expression in a number of OCCC cell lines with various mutational signatures (ES2 and TOV21G, Table 7) as well as two T‐antigen immortalized OSE cell lines (OSE1 and OSE3). DHX38/PRP16 knockdown using lentivirally delivered shRNAs resulted in a strong inhibition of cell viability in OCCC cells, but not in OSE cells (Fig. 2A). DHX38/PRP16 knockdown using these shRNAs was confirmed at both the transcriptional and translational level (Fig. 2B,C).

**Table 7:** Mutational profiles of OCCC cell lines.

OCCC cell line name	Mutations^a^
JHOC5	None
ES2	TP53, BRAF, JAK1
TOV21G	PIK3CA, ARID1A, ARID1B, KRAS, PTEN, CTNNB1, JAK1

^a^ The Cancer Cell Line Encyclopedia (CCLE) was used to identify mutations common to OCCC (ARID1A, ARID1B, SMARCA4, PIK3CA, PTEN, TP53, KRAS, BRAF, CTNNB1, TERT, PP2R1A, JAK, STAT3) in each OCCC cell line.

Recently, DHX38/PRP16 has been implicated in the proliferative potential of the HCT116 colorectal carcinoma cell line, where cells harboring an active KRAS mutant become responsive to DHX38/PRP16 knockdown [26]. However, as reported in the Cancer Cell Line Encyclopedia, of the three OCCC cell lines employed in the current study, ES2 and JHOC5 have no activating mutations in the KRAS signaling pathway. This suggests that while DHX38/PRP16 growth dependency in colorectal carcinoma cells may rely on KRAS, the link between the two in other cancers, including OCCC, may not be as strong.

### DHX38/PRP16 knockdown in OCCC induces apoptosis

We next wondered whether DHX38/PRP16 knockdown might be inducing apoptosis in knockdown‐susceptible cell lines. Cells were transfected with either a control siRNA or one of two siRNA‐targeting DHX38/PRP16 and allowed to proliferate for 4 days, after which DHX38/PRP16 knockdown was confirmed at both the transcriptional and translational level (Fig. 3A,B). We employed RT‐qPCR to determine whether we could observe any change in the transcript level of several pro‐apoptotic genes [p53 upregulated modulator of apoptosis (PUMA), growth arrest and DNA damage‐inducible alpha (GADD45A), NOXA, BAX, BID, IL6, and TNFA] upon DHX38/PRP16 knockdown. We found the potent pro‐apoptotic factor PUMA to be transcriptionally upregulated in a cancer‐specific manner (Fig. 3C), suggesting possible apoptotic induction by p53. We also detected transcriptional upregulation of GADD45A (Fig. 3D), which is also known to play a role in the induction of apoptosis.

To corroborate these results, we employed propidium iodide staining of OCCC and OSE cells 4 days post‐DHX38/PRP16 knockdown using lentivirally delivered shRNAs followed by flow cytometry. DHX38/PRP16 knockdown resulted in a sharp (2–10×) increase in the sub‐G1 population compared to a cohort expressing shLuc in OCCC cells; we were unable to observe a similar increase in OSE cells (Fig. 3E). Interestingly, although the ES2 OCCC cell line harbors a mutation in one allele of p53, it still shows a level of growth inhibition and apoptotic induction upon DHX38/PRP16 knockdown similar to that of p53 nonmutant OCCC lines [27].

To help elucidate the potential role of p53 in DHX38/PRP16 knockdown‐mediated apoptosis, we employed two HCT116 cell lines, one with wild‐type p53 (HCT116‐p53^wt/wt^), and one with a p53 double knockout (HCT116‐p53^−/−^). Knockdown of DHX38/PRP16 via RNAi led to a concomitant decrease in cell viability in both HCT116‐p53^wt/wt^ and HCT116‐p53^−/−^ cells (Fig. 4A,B). Additionally, DHX38/PRP16 knockdown led to an increase in PUMA expression, as well as to an increase in cleaved caspase‐3 levels in HCT116‐p53^wt/wt^ cells (Fig. 4C). This effect was much less pronounced in HCT116‐p53^−/−^ cells. Even so, as stated above, HCT116‐p53^−/−^ cells show a similar level of growth inhibition to HCT116‐p53^wt/w^ upon DHX38/PRP16 knockdown. This suggests that while the cell growth inhibition seen upon DHX38/PRP16 knockdown may proceed in part through the p53 pathway in cells that have an intact pathway, it is not strictly requisite for such growth inhibition.

Overall, the above data provide evidence suggesting that the induction of apoptosis brought about by DHX38/PRP16 knockdown is not necessarily dependent on the presence of wild‐type p53, but that it instead proceeds through multiple p53‐dependent and p53‐independent pathways. In the context of OSE, this would suggest that the lack of growth inhibition seen following DHX38/PRP16 knockdown may not be due to the presence of the large‐T antigen and therefore that knockdown of DHX38/PRP16 may result in an OCCC‐specific induction of apoptosis.

### DHX38/PRP16 knockdown abrogates the tumorigenicity of OCCC cell lines

Finally, in order to confirm the applicability of DHX38/PRP16 impairment to the treatment of OCCC, we sought to replicate our findings *in vivo*. We established TOV21G and ES2 cell lines constitutively expressing either one of two shRNAs targeted to DHX38/PRP16 or to the luciferase gene and implanted them into the flanks of immunocompromised mice. Mice implanted with TOV21G (Fig. 5A) or ES2 (Fig. 5B) cells infected with a control shRNA expression cassette against the luciferase gene (shLuc) all developed tumors, whereas mice implanted with shDHX38/PRP16‐expressing cells showed either significantly impaired or no tumor development. Additionally, the average tumor mass of the shLuc cohort upon completion of the study was found to be significantly greater than either of the two shDHX38/PRP16 cohorts for both TOV21G (Fig. 5C) and ES2 (Fig. 5D). These results confirm *in vivo* that DHX38/PRP16 expression is critical for the growth of OCCC cells.

## Discussion

The present study highlights the importance of DHX38/PRP16 for the tumorigenicity of OCCC; its knockdown induces apoptosis *in vitro* and prevents tumor formation *in vivo*. We found that DHX38/PRP16 knockdown led to an OCCC cell‐specific transcriptional upregulation of GADD45A, a target of p53 and BRCA1 which is known to play roles in DNA repair and cell cycle checkpoint control, as well in the induction of apoptosis [28]. The transcriptional upregulation of PUMA observed upon DHX38/PRP16 knockdown in not only OCCC cells but also HCT116‐p53^wt/wt^ cells would indeed support a role for p53 in DHX38/PRP16 knockdown‐induced apoptosis.

We also were able to observe PUMA upregulation upon DHX38/PRP16 knockdown in HCT116‐p53^−/−^ cells, though at a reduced level compared to HCT116‐p53^wt/wt^ cells. As both post‐knockdown p53^wt/wt^ cells and p53^−/−^ cells showed a similar degree of growth inhibition, this would imply the involvement of one or several additional apoptotic pathways unrelated to p53. PUMA, while most frequently associated with its role in apoptosis directly downstream of p53, can also be upregulated by a number of unrelated transcription factors independent of p53 activity [29, 30]. In one study, NF‐κB was identified as being directly responsible for PUMA upregulation in an HCT116‐p53^−/−^ cell line in response to treatment with TNF‐α [31]. Certain proteasome inhibitors have also been found to activate PUMA and lead to apoptosis in a p53‐independent manner [32]. Taken together, this hints at the possibility that post‐DHX38/PRP16 apoptotic induction is occurring partially independent of p53. Future research will focus on elucidating the p53‐dependent and p53‐independent mechanisms underlying DHX38/PRP16 knockdown‐induced apoptosis in OCCC.

Consistent with our result, amplification of DHX38/PRP16 has been found in as many as 56% of acute myeloid leukemia specimens as well as in established acute myeloid leukemia cell lines [33]. Additionally, several families of splicing factors, including the DDX/DHX family of RNA helicases to which DHX38/PRP16 belongs, have long been implicated in tumor progression and cellular proliferation [24, 34, 35, 36, 37, 38, 39]. Our future research will therefore also focus on elucidating the splicing‐related mechanisms through which DHX38/PRP16 may play a role in the induction of apoptosis upon knockdown in OCCC.DHX38/PRP16 has also been found to have roles outside of the spliceosome; for example, it binds to the protein phosphatase 4 (PP4) protein phosphatase complex, inhibiting the dephosphorylating activity of its PP4C/PP4R2 subunits in both cancer and normal cell lines [40]. Thus, we will also need to consider what mechanisms other than mRNA splicing may be at play.

## Conflict of interest

The authors declare no conflict of interest.

## Author contributions

BC, TH, and TA designed experiments; BC, TH, AY, and NS performed experiments; NY, RN, and KS analyzed sequencing data; BC, TH, and TA analyzed experiments and wrote the manuscript.

## Data Availability

The data that support the findings of this study are openly available in NCBI's Gene Expression Omnibus at https://www.ncbi.nlm.nih.gov/geo/query/acc.cgi?acc=GSE188485, reference number GSE188485. Code for computational analyses is available from the Lead Contact, Tetsu Akiyama (akiyama@iqb.u-tokyo.ac.jp).
